# Literacy and Cognitive Function in the Context of Marriage: A Study of Older Mexican Couples

**DOI:** 10.1093/geroni/igaa057.3168

**Published:** 2020-12-16

**Authors:** Joseph Saenz

**Affiliations:** University of Southern California, Los Angeles, California, United States

## Abstract

Many studies have reported that literacy is associated with favorable cognitive outcomes in late-life. Few have evaluated whether the cognitive benefits of literacy extend to a spouse’s cognitive ability. Among married husband-wife dyads from the 2012 Mexican Health and Aging Study (n=4,078 dyads), literacy was assessed as self-reported ability to read and write. General cognitive ability was assessed using performance across several cognitive domains. Approximately 11% and 15% of husbands and wives, respectively, could not read or write. For both husbands and wives, both own literacy, and having a spouse who could read and write were independently associated with better cognitive ability even after accounting for both partners’ education. Literacy may represent an important form of capital that may be beneficial to preserve cognitive function among older adults. Benefits of spousal literacy may operate by facilitating access to resources such as information and cognitive stimulation.

